# Motor Coordination Correlates with Academic Achievement and Cognitive Function in Children

**DOI:** 10.3389/fpsyg.2016.00318

**Published:** 2016-03-15

**Authors:** Valter R. Fernandes, Michelle L. Scipião Ribeiro, Thais Melo, Paulo de Tarso Maciel-Pinheiro, Thiago T. Guimarães, Narahyana B. Araújo, Sidarta Ribeiro, Andréa C. Deslandes

**Affiliations:** ^1^School of Sports and Physical Education, Federal University of Rio de JaneiroRio de Janeiro, Brazil; ^2^Center for Alzheimers Disease and Related Disorders, Institute of Psychiatry, Federal University of Rio de JaneiroRio de Janeiro, Brazil; ^3^Institute of Physical Education - Federal University FluminenseRio de Janeiro, Brazil; ^4^Exercise Physiology Laboratory, University Estácio de SáRio de Janeiro, Brazil; ^5^Brain Institute, Federal University of Rio Grande do NorteNatal, Brazil; ^6^Neuroscience Exercise Laboratory, Instituto de Educação Física e Desporto da Universidade Estadual do Rio de JaneiroRio de Janeiro, Brazil

**Keywords:** motor skills, child, educational status, physical exercise, executive functions

## Abstract

The relationship between exercise and cognition is an important topic of research that only recently began to unravel. Here, we set out to investigate the relation between motor skills, cognitive function, and school performance in 45 students from 8 to 14 years of age. We used a cross-sectional design to evaluate motor coordination (Touch Test Disc), agility (Shuttle Run Speed—running back and forth), school performance (Academic Achievement Test), the Stroop test, and six sub-tests of the Wechsler Intelligence Scale for Children-IV (WISC-IV). We found, that the Touch Test Disc was the best predictor of school performance (*R*^2^ = 0.20). Significant correlations were also observed between motor coordination and several indices of cognitive function, such as the total score of the Academic Achievement Test (AAT; Spearman's rho = 0.536; *p* ≤ 0.001), as well as two WISC-IV sub-tests: block design (*R* = −0.438; *p* = 0.003) and cancelation (rho = −0.471; *p* = 0.001). All the other cognitive variables pointed in the same direction, and even correlated with agility, but did not reach statistical significance. Altogether, the data indicate that visual motor coordination and visual selective attention, but not agility, may influence academic achievement and cognitive function. The results highlight the importance of investigating the correlation between physical skills and different aspects of cognition.

## Introduction

Regular physical activity is well-known to promote several positive changes in health, including cardio respiratory benefits, increased bone mineral density, and decreased risk of chronic degenerative diseases (Garber et al., 2011). Exercise improves several physical fitness parameters comprising a set of measurable health and skill-related attributes, such as cardio-respiratory fitness, muscular strength and endurance, body composition, and flexibility. These health-related components of physical fitness are recognized to be very important for public health (Garber et al., 2011). Not least, agility and motor coordination are physical attributes directly related to sports and daily activities (Caspersen et al., 1985). Motor coordination comprises the harmonization of the nervous and musculoskeletal systems, resulting in a rapid, accurate, and balanced motor response, normally assessed by measurements of hand-eye or foot-eye coordination (Corbin et al., 2000; Lopes et al., 2012). Agility relates to the ability of rapidly changing the position of the entire body in space with speed and accuracy (Singh, 2013). The proficiency in these skill-related aspects of physical fitness in childhood may predict an active lifestyle during adolescence (Barnett et al., 2009). In a recent systematic review, Van der Fels and collaborators showed of a relationship between cognition and certain motor skills. Among a selected set of 21 articles, bilateral body coordination showed strong relationship with fluid intelligence, whereas fine motor skills presented a moderate to strong relationship with visual processing, two cognitive skills highly required in complex motor tasks (van der Fels et al., 2015). In pre-schoolers, an evaluation of data sets from three longitudinal studies has found that fine motor skills are a strong predictor of later reading and math achievement (Grissmer et al., 2010). In this context, early motor development seems to both require and enhance a sophisticated cognitive capacity, later used throughout school life (Grissmer et al., 2010).

Besides, promoting physical fitness and metabolic health, physical exercise can contribute to the improvement of specific cognitive functions in adults (Masley et al., 2009) as well as children (Chaddock et al., 2011; Diamond and Lee, 2011). Among the cognitive benefits of an active lifestyle, it seems that physical exercise may specifically benefit executive functions (EFs; Hillman et al., 2008), which comprise inhibitory control, planning, working memory, decision making, and cognitive flexibility (Miyake et al., 2000). More specifically, core executive functions are Inhibition, working memory, and cognitive flexibility (Diamond, 2013). These cognitive functions are required for the performance of daily activities, being particularly important for cognitive and motor development (Miyake et al., 2000), and social and emotional relationships throughout life (Moffitt et al., 2011). Working memory is essential for learning, inhibitory control is critical for attention, and both processes are directly related to academic achievement (Redick and Engle, 2006; Martinussen and Major, 2011; Haapala et al., 2014). Among the different regions of the brain that are involved in EFs, the prefrontal cortex (PFC) is the one with the slowest development (Quartz and Sejnowski, 1997). Several studies have demonstrated how this prolonged development makes the PFC especially susceptible to the influence of physical activity and exercise throughout life (Halperin and Healey, 2011; Mackey et al., 2012).

Neuroimaging studies indicate, that some of the brain regions previously thought to be exclusively related to motor activity (cerebellum and basal ganglia) or cognition (PFC) are co-activated during the execution of specific cognitive or motor activities (Diamond, 2000). Neuronal connections link the PFC and the cerebellum, which together with the basal ganglia are directly involved in the control of coordinative exercises (Budde et al., 2008). Physical exercise increases cerebral blood volume (Pereira et al., 2007) and basal ganglia volume (Chaddock et al., 2010), and promotes the release of neurotransmitters, (e.g., norepinephrine, dopamine, and serotonin), and trophic factors, such as the brain derived neurotrophic factor (Dishman et al., 2006). These molecular responses to physical exercise promote synaptogenesis, angiogenesis, and neurogenesis specifically in the hippocampus, as has been shown in rodents (Van Praag et al., 1999; Fabel et al., 2009), and suggested by the increased hippocampal volume in humans (Erickson et al., 2011). Indeed, physical exercises have been proposed to contribute to an improvement of EFs, to the academic performance of children and to a greater activation of the PFC (Davis et al., 2011). Moreover, higher levels of aerobic fitness are related to a greater capacity for inhibitory control (Buck et al., 2008). Studies with school-age children found a positive correlation between maximal cardiorespiratory fitness (VO_2max_), cognitive control, selective attention and visual memory, which was associated with increased volume of the basal ganglia and the activation of the prefrontal and parietal cortices (Chaddock et al., 2012). The dorsal striatum appears to be specifically involved in the control of cognitive responses, which may be positively influenced by aerobic fitness (Chaddock et al., 2010). Indeed, even a single session of aerobic exercise can facilitate children's cognitive performance (Hillman et al., 2009).

Although, the majority of the studies have investigated the relationship between aerobic training and cognitive function, other types of physical exercise may also be associated with positive cognitive and academic impacts. Studies investigating the effect of Tae-kwon-do showed a reduction in aggression, improvement in emotional control, self-esteem, social life, and school performance (Trulson, 1986; Lakes and Hoyt, 2004). Improvement in emotional control can contribute to better school performance. In this regard, a longitudinal study of 1000 individuals for 30 years highlighted emotional control as a good predictor of school performance, social, emotional, and economic status (Moffitt et al., 2011). In addition, activities that include meditation and breathing exercises, such as Tai Chi and Yoga, showed positive effects on attention, planning, and emotional control of school children (Manjunath and Telles, 2001; Wall, 2005; Flook et al., 2010).

Coordinative exercises and motor coordination also seem to be related to EFs. Budde and collaborators verified, that 10 min of acute bilateral coordination exercises promoted more improvement in concentration and attention of school children than a normal physical education lesson with the same duration (Budde et al., 2008). Since, the heart rate was not significantly different between both groups, it is possible that the coordinative characteristic of the exercises was responsible for the results (Budde et al., 2008). In overweight children, a physical education program that involved cognitively challenging tasks and open skills activities, characterized by an unstable environment demanding continuous adaptation, was able to enhance inhibitory control (Crova et al., 2013). Likewise, a meta-analysis of performance in inhibitory control tasks yielded better results in athletes than in non-athletes (Erickson et al., 2009). Those benefits seem to be cumulative. In a 9 year-long intervention study, physical education classes with increased time and intensity were associated with better school performance than normal-intensity or low-intensity classes (Ericsson and Karlsson, 2012). Not coincidentally, the neural circuits recruited by motor coordination and executive attention comprise the PFC, cerebellum, and anterior cingulate cortex (Bush, 2000; Diamond, 2000). This network is linked to the hippocampus through the anterior cingulate cortex, and influences the learning process, especially in the consolidation of new memories (Posner and Rothbart, 2014).

A better understanding of the influence of skills such as coordination and agility on school learning can contribute to design more efficient physical exercise programs capable to promote not just physical and social benefits, but also enhance children's cognition. At present, there is a lack of studies that investigate the relationship between motor coordination, agility, EFs and academic achievements. To address this gap, we assessed the correlations linking motor skills to EFs and academic achievement in children. The current study aimed to investigate the relationship between motor coordination, agility, EFs, and academic achievements.

## Methods

### Participants

The sample of this cross-sectional study consisted of 45 children from 8 to 14 years of age, literate, of both sexes, from the municipal school Compositor Luiz Gonzaga, located in the municipality of Rio de Janeiro, Brazil. This school presents high prevalence of educational backwardness and functional illiteracy, with a large age range in the same class. The participants were recruited from the fourth or fifth years of elementary school. The exclusion criteria were functional illiteracy, attention deficit hyperactivity disorder, intellectual disabilities, visual, or hearing impairment without correction, and inability to participate in physical assessments. The participants were evaluated in 4 stages, in the following order: Signature of forms, neuropsychological assessment (WISC-IV sub-tests and Stroop test); school performance (Academic Achievement Test); and physical assessment (anthropometric measurements, motor coordination, and agility tests). The assessments were performed in the school setting, in random order on alternate days. Participants and their parents were informed about the experimental procedure and signed an Informed Consent Form and a Statement of Consent. This project was approved by the Ethics and Research Committee, University Hospital, UFRJ (CAAE 26338114.7.0000.5257).

### Neuropsychological assessment

This stage of evaluation was completed in one session of ~2 h, being extended for two 1-h sessions whenever necessary. Subjects were evaluated by WISC-IV sub-tests and Stroop test.

- WISC-IV: The following sub-tests of WISC-IV were performed in order to assess core EF and other cognitive functions. The score was obtained in each sub-test by summing the weighted scores given to the correct responses (Wechsler, 2013):

1. *Block Design*—The following sub-test is a non-verbal activity that measures the individual's abilities in spatial visualization and analysis, processing, and visual-motor coordination. Block Design is a timed perceptual reasoning sub-test. Children are given blocks with two red sides, two white sides, and two red/white sides, to replicate the modeled design presented by the psychologist or printed in the application book;
2. *Similarities*—Two different words are verbally presented, and the student is asked to say how (and if) the meanings of both words are similar. This sub-test measures verbal concept formation and verbal abstract thinking;
3. *Digit forward*—Measures short-term memory and attention. The student must repeat random number sequences in the order of presentation;
4. *Digit backward*—Measures working memory and attention. The student must repeat random number sequences in the reverse order of presentation;
5. *Letter–Number Sequencing*—Measures working memory and attention. The student must repeat the numbers in chronological order, and the letters in alphabetical order, following a presentation in random sequence;
6. *Cancelation*—Measures visual selective attention and processing speed. The child needs to cross out with a pencil as many animals as possible from a set of colorful animals and objects printed in two different sets of two-page spread, in precisely in 45 s. In the first set of pages, the images are organized randomly; the second set is organized vertically and horizontally;

- The *Stroop Test* assess selective attention and concentration, cognitive flexibility and inhibitory control. During each of three conditions (word, color, incongruent color-word), participants were instructed to read out loud, as quickly, and accurately as possible, the color in which different colored words or letters are printed. In the first condition, they had to read colored lists sequences of the letter “X.” In the second condition they had to read lists of various colorful words. In the third and incongruent color-word condition, participants had to read a list of color words written in incongruent color ink relative to the printed word (e.g., the word red printed in blue ink). (Stroop, 1935). The time performed in each condition was written down with a precision of seconds. Stroop delta score is equivalent to the time performed in color-incongruent condition, minus the time performed in word condition.

### School performance

The Academic Achievement Test (AAT) that investigates the fundamental skills for children's school performance, indicating knowledge in writing, reading, and arithmetic, evaluated Participants. First, the ATT assess the child's ability to spell words correctly. Then, in the arithmetic subtest, the examiner verbally presents math problems, in which the child writes down the answers. Finally, for the reading sub-test, the child reads words out loud (Stein, 1994).

### Physical fitness assessment

All participants were evaluated in anthropometric variables to characterize the sample with respect to weight and height, through which we calculated the *body mass index (BMI)*. Afterwards, the BMI were classified as normal, overweight, or obese, by a worldwide children's standard range for BMI (Hammer et al., 1991; Cole et al., 2000) The instruments used were the Vonder Trena, (keychain model) brand with a length of 200 cm and a resolution of 1 mm, set at a flat wall for assessment of stature; Digital scale G-Tech with a precision of 1 kg, and a resolution of 1 kg; Stopwatch Vollo (VL 237 model).

#### Touch test discs (TTD)

**Figure 1:**
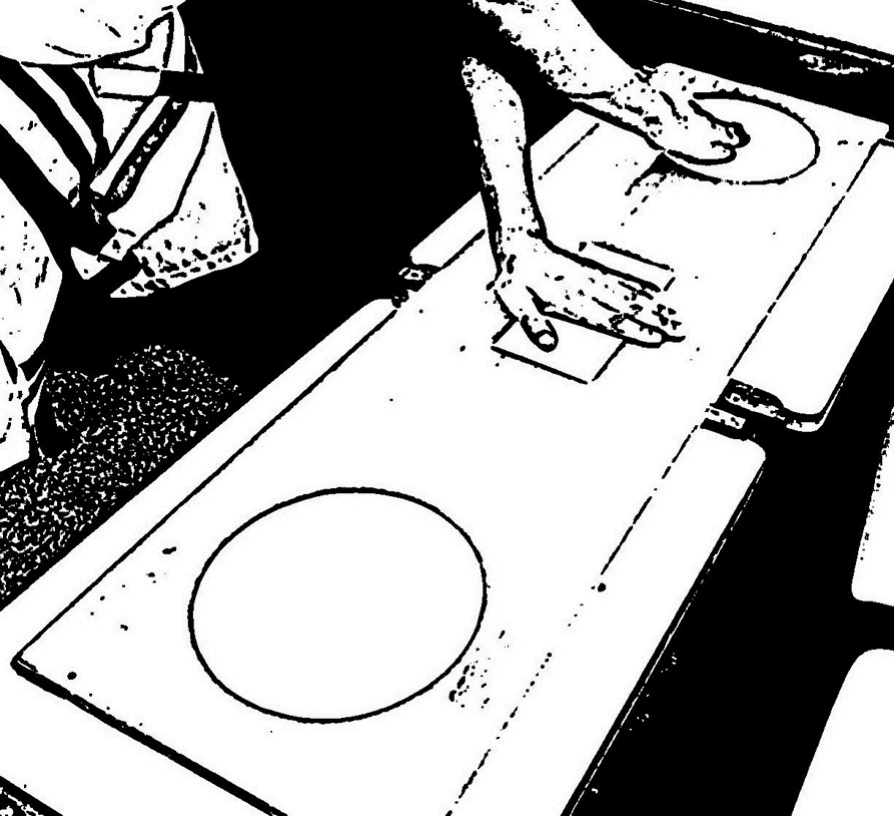
**Touch Test Disc board (TTD)**.

Evaluates Motor coordination, especially hand-eye coordination. The test is performed on a rectangular wooden plank with 120 cm wide by 60 cm wide. In the center of the board, it contains a rectangle of 10 cm high by 20 wide and a circle of 20 cm in diameter on each side with a distance of 5 cm between the figures (Figure 1). The individual has to keep the non-dominant hand in the central rectangle, and touch with the dominant hand in the circle on the opposite side, crossing his arm over the other, and come back to complete one cycle. Each attempt comprises 25 correct cycles, and the smallest time for completion out of three attempts is considered (Gobbi et al., 2005).

#### Shuttle run speed—“running back and forth” test

Evaluates agility that consists in the ability to change the position of the body or movement direction with velocity. For its realization, a space of 5 m is marked with tape on the ground. The subject must perform five cycles, running back, and forth, to complete the test. The result is written down with a precision of tenths of seconds (Gobbi et al., 2005).

### Statistical analyses

The Kolmogorov-Smirnov test was used to evaluate the Gaussianity of the variables. Linear regression was used to analyze the unstandardized coefficient of motor variables predicting school performance (AAT). Moreover, Pearson (parametric) and Spearman (non-parametric) correlation was done to evaluate the correlation between motor and cognitive variables. A Bonferroni correction adjusted the *p*-value in relation to the number of correlations that were performed (*p* ≥ 0.006). A one-way ANOVA was used to compare the dependent variables among groups according BMI (normal × overweight × obese). All statistical analyses were carried out using the SPSS® for Windows, Version 19.

## Results

**Figure 2:**
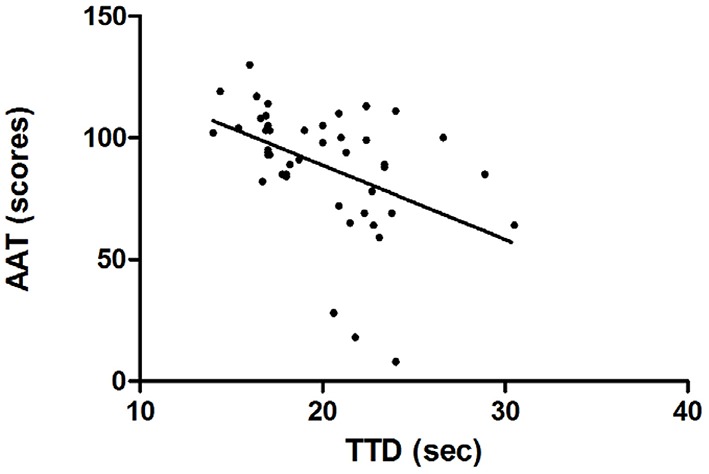
**AAT, Academic Achievement Test**. TTD, Touch Test Disc (*r*_*s*_ = 0.53; *p* ≤ 0.001).

Descriptive analyses are provided in Table 1. The participants had a mean age of 10.40 years, and 57.78% were female. Anthropometric measurements resulted in values, that compose the BMI. The subjects were classified by BMI groups, being 55.56% in normal range by age, 28.89% overweight, and 15.56% obese (Hammer et al., 1991; Cole et al., 2000). There were no significant differences among BMI groups in all cognitive and physical dependent variables as shown in Table 1. Surprisingly neither of the tests that assess specifically the core EFs showed significant correlation with TTD or Agility. Linear regression was used to verify the association between motor variables (TTD and agility test) and school performance (AAT). The results showed that TTD is the best predictor of positive results in school performance (*R*^2^ = 0.20). In agreement with this evidence, a significant correlation between TTD and AAT total was verified (Figure 2). Note, that shorter TTD times are associated with better results in AAT. This suggests a presenting influence of motor coordination in academic performance. TTD also showed a negative correlation with Block design, thus shorter times in TTD indicates higher scores in Block Design, which demands spatial visualization and analysis, processing, and visual-motor coordination (VMC). Moreover, TTD presented a negative correlation with Cancelation, which requires visual selective attention and processing speed, fine-motor coordination (Figure 2). Altogether, the results indicate that shorter times in the motor coordination test were associated with higher academic achievement, and better scores in cognitive tasks related to VMC. In addition, faster times in the agility test correlated with better results in the cognitive tests. Scores in the agility test presented negative parametric correlation with Block Design, Cancelation, and a non-parametric correlation with Digit Forward. However, none of these correlations reached statistical significance after Bonferroni correction for the number of comparisons (Table 2).

**Table 1:** **Descriptive data and values of the physical and cognitive tests categorized by BMI status**.

	**Total *X* (*SD*) Min–Max *N* = 45**	**Normal *X* (*SD*) Min–Max *N* = 25**	**Overweight *X* (*SD*) Min–Max *N* = 13**	**Obese *X* (*SD*) Min–Max *N* = 7**	**ANOVA *F* (*p*)**
Age (years)	10.40 (1.38) 8.00 − 14.00	10.76 (1.362) 8 − 13	10 (1.472) 8 − 14	9.85 (1.069) 9 − 12	2.002 (0.148)
Weight (kg)	42.32 (10.61) 26.00 − 67.00	37.18 (8.528) 26.00 − 50.00	44.30 (7.532) 34.00 − 60.00	57.00 (7.234) 45.00 − 67.00	17.01 (<0.001)
High (cm)	144.00 (0.89) 122.00 − 164.00	143.96 (0.108) 122.00 − 164.00	144.35 (0.064) 133.00 − 156.00	144.29 (0.054) 138.00 − 154.00	0.009 (0.991)
BMI	20.17 (3.92) 14.70 − 30.50	17.68 (1.848) 14.70 − 21.21	21.10 (1.742) 19.22 − 24.65	27.31 (2.482) 23.63 − 29.93	70.618 (<0.001)
TTD (s)	19.99 (3.67) 14.00 − 30.50	19.44 (2.767) 16.00 − 24.00	21.37 (4.176) 14.40 − 30.5	19.40 (5.266) 14.00 − 28.90	1.298 (0.284)
Agility (s)	24.83 (2.10) 21.70 − 32.00	24.55 (1.915) 21.90 − 28.40	25.01 (1.693) 22.60 − 28.70	25.51 (3.336) 21.70 − 32.00	0.627 (0.539)
AAT total	88.75 (25.07) 8.00 − 130.00	88.80 (26.817) 8.00 − 130.00	87.92 (25.782) 18.00 − 119.00	90.14 (20.045) 64.00 − 117.00	0.017 (0.983)
Stroop delta	17.60 (12.04) −4.00 − 54.00	16.08 (10.491) −4.00 − 37.00	21.15 (15.345) 5.00 − 54.00	16.49 (10.706) 4.00 − 29.00	1.236 (0.301)
Digit forward	6.35 (1.24) 2.00 − 9.00	6.44 (1.003) 4.00 − 8.00	5.84 (1.519) 2.00 − 8.00	7.00 (1.291) 6.00 − 9.00	2.193 (0.124)
Digit backward	5.35 (1.62) 1.00 − 10.00	5.56 (1.609) 2.00 − 10.00	5.15 (1.908) 1.00 − 9.00	5.00 (1.154) 4.00 − 7.00	0.454 (0.638)
Cancelation	79.11 (15.49) 53.00 − 116.00	78.68 (15.154) 54.00 − 108.00	78.00 (17.392) 53.00 − 116.00	82.71 (14.817) 63.00 − 99.00	0.224 (0.800)
Block design	18.15 (8.51) 4.00 − 35.00	18.24 (7.468) 4.00 − 35.00	18.61 (10.500) 6.00 − 35.00	17.00 (9.255) 4.00 − 31.00	0.081 (0.922)
Letter − number seq.	11.75 (3.93) 5.00 − 18.00	12.40 (3.796) 6.00 − 18.00	11.69 (3.497) 6.00 − 16.00	9.57 (4.928) 5.00 − 18.00	1.442 (0.248)
Similarities	8.08 (5.08) 1.00 − 23.00	8.48 (5.665) 1.00 − 23.00	8.00 (3.535) 4.00 − 17.00	6.85 (5.814) 1.00 − 18.00	0.272 (0.763)

*X, Mean; SD, standard deviation; min, minimum; max, maximum; BMI, Body Mass Index; TTD, Touch Test Disc, motor coordination test; Cancel, Cancelation; AAT, Academic Achievement Test; Letter–number seq, Letter–number sequencing*.

**Table 2:** **Correlations between physical and cognitive tests**.

	**TTD**	**Agility**
AAT total^a^	−0.536^*^ (<0.001)	−0.154 (0.313)
Stroop delta	−0.071 (0.643)	0.136 (0.373)
Digit forward^a^	−0.150 (0.327)	−0.334 (0.025)
Digit backward^a^	−0.238 (0.115)	−0.201 (0.186)
Cancelation	−0.471^*^ (0.001)	−0.301 (0.044)
Block design	−0.438^*^ (0.003)	−0.282 (0.060)
Letter−number sequencing	0.351 (0.018)	−0.260 (0.085)
Similarities^a^	0.006 (0.970)	−0.004 (0.980)

a *Spearman's rho; AAT, Academic Achievement Test*;

* *Significant results (≥0.006 after Bonferroni correction for multiple comparisons)*.

## Discussion

The findings of the present study suggest that a specific aspect of motor skills, namely motor coordination, is directly related to the academic achievement and among the variables investigated, the best predictor was motor coordination evaluated by TTD. Contrary to our expectation, neither of the tests that assess specifically the core EFs (*Number and Letter Sequence, Digit forward, Digit Backward*, and *Stroop test)* showed significant correlation with TTD or Agility. Moreover, agility does not associate with academic achievement and cognitive skills. These results corroborate a recent systematic review about relationship between cognitive and motor skills in children (van der Fels et al., 2015). The authors showed best results for the relationship between cognitive skills and complex motor skills (fine motor skills, bilateral body coordination, and timed performance) than balance, strength and agility that seems to require less cognitive effort. In particular, fluid intelligence, and visual processing, two cognitive skills highly required in complex motor tasks, presented more significant correlations.

The Block design and Cancelation tests were also related to better results in TTD. In those tests, students must use fine motor coordination and selective visual attention. In addition, specific aspects of EFs, visuospatial working memory and inhibitory control are indirectly activated to retrieve images and suppress impulsive responses during the tests (Baddeley, 2003; Diamond, 2013). These abilities are essential for writing in particular and for learning in general, and are directly related to academic achievement (Grissmer et al., 2010). These results can be explained by the neuronal connections involved in coordinative exercises (CE) and visual motor capacity. Cerebellum, parietal posterior cortex (PPC), and PFC are involved in motor and executive functions, mediating a variety of neurocognitive processes, such as working memory, attention, perception, and verbal learning. This network is specially activated, when performing a complex motor task, a new task or a condition of task change, a quick reply is requested, and attention is needed to perform the task. Other hypothesis is that motor and cognitive skills might have a similar developmental timetable, with a development peak between 5 and 10 years of age. Also, motor and cognitive activities, require various common underlying processes, such as sequencing, monitoring, and planning (van der Fels et al., 2015). CE promotes activation of these areas, which facilitate not only a greater accuracy in motor performance, but also the cognitive functions controlled by them (Budde et al., 2008; Westendorp et al., 2014).

The results of the present study are in general agreement with the literature. Research by Carlson and colleagues showed a positive correlation between VMC and visual spatial integration (VSI), with mathematical and written expression. In the VMC test, subjects had to trace geometric forms, while in the VSI test they had to copy a geometric form, which requires not just the control of small muscle movement, but also a mental representation of an image and its replication (Carlson et al., 2013). In our study, Block Design required the representation of an image, to be replicated through the manipulation of the blocks, in a task similar to VSI. Furthermore, TTD demands accurate motor control and visual attention, similarly to VMC.

Interventional studies with physical activities that contained complex motor and cognitive challenges also have shown positive effects on cognition. Budde and collaborators showed that acute CE was more efficient in enhancing concentration and attention than normal sport lessons (Budde et al., 2008). There was no significant difference in the intensity between the groups, which assures that CE was specifically relevant for the results. Even among kindergarten children, CE might promote positive effects in EFs, such as a higher response accuracy and shorter reaction times (Chang et al., 2013). A recent study presented evidence that not just physical fitness, but also motor and sport skills can predict EF performance in adolescents (Marchetti et al., 2015). A meta-analysis review of cognition and sport expertise demonstrated that expert athletes performed better on processing speed and attentional domains than non-expert athletes (Voss et al., 2009). Altogether, these results highlight the importance of motor coordination proficiency for a healthy cognitive performance.

The correlation between motor coordination and school achievement, verified in this study, seems to be related to motor ability and visual perception required for object identification and localization. Action systems (Jeannerod, 2006) and anticipatory systems (Akshoomoff et al., 1997) involve different brain regions related to attentional control, visual processing (cerebellum, dorsolateral prefrontal cortex, parietal posterior cortex, middle occipital, and inferior temporal cortices), and specific brain regions related to response selection and planning (anterior cingulate cortex, supplementary motor areas, and precuneus; Liu et al., 2004). Several studies indicate, that the deficit in visuospatial attention is related to motor and cognitive deficits found in children with developmental coordination disorder (Tsai, 2009). Altogether, these studies point to the importance of coordinative exercises during schooling.

There are some limitations that need to be considered. First of all, BMI had a large variability between subjects. However, there was no significant difference between BMI groups among dependent variables. Another limitation is the lack of information about children maturation, since there was a large age span (8–14). Future research should investigate the relationship between different complex motor skills (fine motor skills, bilateral body coordination, hand-eye coordination, and foot-eye coordination) with timed performance, allowing evaluation of speed, attention, and quality of performance in the tasks. Shall also, evaluate the influence of physical skills related to sports on cognition and academic achievement, with interventional randomized control trials, comparing the effects of purely aerobic exercises with activities with emphasis in motor coordination, able to promote a wide range of physical abilities, (e.g., capoeira, dance, acrobatics). Such design would lead to a better understanding of which amount and type of exercise can promote the best effects on cognition. The contemporary “epidemic” of child obesity, the need to find new ways to enhance school performance, and all the evidence pointing to the relationship between physical fitness and cognitive performance, highlight the importance of new studies in this area. The present study concluded that visual motor coordination and visual selective attention, but not agility, might influence academic achievement and cognitive function.

## Author contributions

All authors listed, have made substantial, direct and intellectual contribution to the work, and approved it for publication.

### Conflict of interest statement

The authors declare that the research was conducted in the absence of any commercial or financial relationships that could be construed as a potential conflict of interest.
